# Supplementary material for: Effects of different proportions of organic substitution for mineral fertilizers on soil methanogenic and methanotrophic communities in paddy fields
Source: PeerJ. 2025 Mar 26;13:e19000. doi: 10.7717/peerj.19000 (PMC11954458; doi:10.7717/peerj.19000)
Supplement: Supplemental Information 1 [file peerj-13-19000-s001.docx]

**Supporting Information**

**Supporting Materials and Methods**

**Microcosm experiment**

The supplementary materials comprise the detailed information of this section. A gas chromatograph (GC, Agilent 7890B, USA) is equipped with a flame ionization detector (FID) for determining CH_4_ concentration. The formula for calculating CH_4_ emission flux is:

$$F= \frac{M}{V_{0}}\times\frac{V}{m}\times\frac{d_{c}}{d_{t}}\times\frac{T_{0}}{T}\times\frac{P}{P_{0}}$$

where: F is the gas flux (μg·kg^-1^h^-1^); M is the molar mass of the gas (g·mol^-1^); *V*_0_ is the molar volume of an ideal gas under standard conditions (22.41 L·m^-3^). *V* and *m* are effective gas volume (m^3^) and soil mass (kg), respectively. *d_c_*/*d_t_* is the change rate of gas mass concentration in the flask per unit time (ppm min^-1^). *T*_0_ is the absolute temperature of the gas in the standard state (*T*_0_ = 273 K), and *P*_0_ is the density of the gas in the standard state (*P*_0_ = 1013 mb). *T* is the actual temperature of the air inside the incubator (K); *P* is the actual local atmospheric pressure (mb).

The formula for calculating cumulative gas emissions is as follows:

$$C_{t}'=C_{t}+\frac{\left( F_{t}+F_{t}^{'} \right)}{2}\times(t^{'}-t)$$

where *C_t_*' and *C_t_* are the cumulative release of CH_4_ (μg·kg^-1^) at *t*' and *t*, respectively. *F_t_*' and *F_t_* are the methane release rates at *t*' and *t*, respectively (μg·kg^-1^·d^-1^), and *t* is the incubation time (days).

**Analysis of soil chemical properties**

Soil pH was determined by measuring a soil–water suspension (1:5 wt/vol) after shaking for 30 min. Total phosphorus (TP) and total potassium (TK) were measured by molybdate calorimetry and flame photometry, respectively (*Li et al. 2023*). Specifically, soil samples were treated with NaOH solution, ensuring all phosphorus-containing minerals and organophosphorus compounds were converted into soluble M_3_PO4, and potassium was rendered soluble; then, an autoAnalyser3 (Bran + Luebbe; Hamburg, Germany) was used to determine them. Soil samples underwent rigorous high-temperature treatment using concentrated H_2_SO_4_ solution (>70%) and mixed catalysts to determine TN. This process hydrolyzed and oxidized nitrogen-containing compounds, resulting in the release of NH_3_, which was quantitatively measured for determination. Available N (AN) content was measured using an alkaline hydrolysis diffusion method (*Cornfield 1960; Jackson 1979*), which quantifies AN by converting easily hydrolyzable organic nitrogen into NH_3_ in soil samples. The available phosphorus (AP) content was determined using the molybdenum blue method (*Olsen et al. 1954*), which is extracted using a sodium bicarbonate solution to isolate adsorbable phosphorus from the soil, followed by quantification using molybdenum antimony spectrophotometry. Available potassium (AK) was measured using flame photometry (*Chen et al. 2020*). The soil organic carbon (SOC) content was determined using the K_2_Cr_2_O_7_-H_2_SO_4_ oxidation method (*Wang et al. 2003*).

**References:**

Chen X, Li T, Lu D, Cheng L, Zhou J, and Wang H. 2020. Estimation of soil available potassium in Chinese agricultural fields using a modified sodium tetraphenyl boron method. *Land Degradation and Developmen* 31:1737-1748. <https://doi.org/10.1002/ldr.3535>

Cornfield AH. 1960. Ammonia released on Treating Soils with N Sodium Hydroxide as a Possible Means of predicting the Nitrogen-supplying Power of Soils. *Nature* 187:260-261. https://doi.org/10.1038/187260a0

Jackson ML. 1979. Soil chemical analysis - advanced course.

Li ZR, Luo SQ, Peng YJ, Jin CZ, and Liu DC. 2023. Effect of long-term application of bioorganic fertilizer on the soil property and bacteria in rice paddy. *AMB Express* 13:60. https://doi.org/10.1186/s13568-023-01559-2

Olsen, Sterling R., C. V. Cole, Frank S. Watanabe, L. A. Dean, and United States Department of Agriculture. 1954. Estimation of available phosphorus in soils by extraction with sodium bicarbonate. Washington, D.C.: U.S. Dept. of Agriculture.

Wang S, Tian H, Liu J, and Pan S. 2003. Pattern and change of soil organic carbon storage in China: 1960s–1980s. *Tellus B* 55:416-427. <https://doi.org/10.1034/j.1600-0889.2003.00039.x>

**Raw Data:**

**Raw data of annual production of grain and straw**

|  | **Annual grain production (kg/ha)** | **Annual straw production (kg/ha)** |
| --- | --- | --- |
| CK | 4295.83 | 5437.02 |
|  | 5210.45 | 5464.60 |
|  | 6285.27 | 5521.56 |
|  | 6791.62 | 5703.21 |
|  | 6746.49 | 5734.31 |
|  | 6786.76 | 5717.8 |
|  | 7202.14 | 5743.81 |
|  | 7102.01 | 5736.46 |
|  | 7242.32 | 5760.99 |
| NPK | 9550.49 | 11393.51 |
|  | 9855.30 | 11321.35 |
|  | 9765.68 | 11285.37 |
|  | 10071.27 | 11042.16 |
|  | 10166.39 | 11060.11 |
|  | 10251.25 | 11083.06 |
|  | 10605.65 | 10364.33 |
|  | 10648.54 | 10288.9 |
|  | 10517.88 | 10207.25 |
| M30 | 11010.01 | 11531.26 |
|  | 11210.15 | 11493.63 |
|  | 11276.13 | 11432.31 |
|  | 11525.51 | 10945.61 |
|  | 11495.42 | 10901.38 |
|  | 11540.56 | 10915.12 |
|  | 12765.75 | 10346.37 |
|  | 12897.77 | 10350.19 |
|  | 12790.93 | 10164.53 |
| M50 | 10628.15 | 11826.81 |
|  | 10662.85 | 11701.64 |
|  | 10854.9 | 11790.88 |
|  | 11896.35 | 10940.99 |
|  | 11847.92 | 10892.82 |
|  | 11867.85 | 10998.78 |
|  | 12663.55 | 10745.65 |
|  | 12613.42 | 10561.86 |
|  | 12631.67 | 10495.48 |
| M70 | 10539.15 | 11722.92 |
|  | 10641.88 | 11500.79 |
|  | 10870.62 | 11581.64 |
|  | 11525.56 | 11261.42 |
|  | 11456.46 | 11312.51 |
|  | 11496.86 | 11414.42 |
|  | 13428.26 | 10985.86 |
|  | 13267.47 | 10702.15 |
|  | 13619.73 | 10824.60 |

**Raw data of total methane (CH₄) emissions**

|  | **Total CH_4_ emission**  **(μg kg^-1^)** |
| --- | --- |
| CK | 4.270463192 |
|  | 6.274333578 |
|  | 6.052458368 |
|  | 6.368668111 |
|  | 5.239218121 |
|  | 4.313167824 |
|  | 6.337076914 |
|  | 6.112982952 |
|  | 6.432354781 |
| NPK | 5.372058753 |
|  | 7.259814912 |
|  | 9.580697987 |
|  | 6.519384753 |
|  | 5.798355076 |
|  | 5.427794983 |
|  | 7.321763061 |
|  | 9.647403596 |
|  | 6.573928600 |
| M30 | 6.645138633 |
|  | 7.181617659 |
|  | 7.867990667 |
|  | 12.03380757 |
|  | 10.30591852 |
|  | 6.729640019 |
|  | 7.286483836 |
|  | 7.909720574 |
|  | 12.17234565 |
| M50 | 13.10087122 |
|  | 10.83037731 |
|  | 7.735785025 |
|  | 8.947068582 |
|  | 9.316716862 |
|  | 13.24957994 |
|  | 10.93938108 |
|  | 9.410842875 |
|  | 8.428239268 |
| M70 | 7.189702915 |
|  | 7.381065751 |
|  | 8.755365877 |
|  | 9.807861472 |
|  | 9.078595008 |
|  | 7.261699944 |
|  | 7.454976408 |
|  | 8.843019535 |
|  | 9.906040087 |

**Raw data of gene copy number**

|  | ***mcrA* gene copy number**  **(copies/g)** | ***pmoA* gene copy number**  **(copies/g)** |
| --- | --- | --- |
| CK | 257.204542 | 490.095691 |
|  | 270.165177 | 256.558814 |
|  | 149.765039 | 397.801889 |
|  | 270.165177 | 248.437844 |
|  | 402.413652 | 550.214097 |
|  | 568.300894 | 1271.320396 |
|  | 395.049191 | 663.398844 |
|  | 315.713984 | 726.391092 |
|  | 241.587704 | 621.125179 |
| NPK | 942.621690 | 2381.083235 |
|  | 2247.664681 | 3206.554450 |
|  | 1772.942476 | 2758.618916 |
|  | 327.859586 | 1069.808067 |
|  | 505.850847 | 1395.588287 |
|  | 548.294696 | 1607.859231 |
|  | 722.110889 | 1541.621631 |
|  | 722.110889 | 1541.621631 |
|  | 722.110889 | 1541.621631 |
| M30 | 4581.582284 | 3982.479601 |
|  | 3706.346664 | 3982.479601 |
|  | 3503.803689 | 2666.782635 |
|  | 3820.629043 | 3982.479601 |
|  | 2073.648298 | 1983.321951 |
|  | 2120.954049 | 1286.470930 |
|  | 2562.969587 | 3982.479601 |
|  | 2562.969587 | 951.958826 |
|  | 1755.211876 | 5251.918750 |
| M50 | 3837.495652 | 4055.365078 |
|  | 4297.252198 | 5127.915778 |
|  | 3614.091007 | 8486.664290 |
|  | 2722.783013 | 4927.236034 |
|  | 3614.091007 | 2390.247683 |
|  | 2926.061578 | 4764.891397 |
|  | 5356.102134 | 8486.664290 |
|  | 5760.799373 | 11038.837101 |
|  | 4980.215343 | 8486.664290 |
| M70 | 5962.463992 | 38328.487083 |
|  | 5962.463992 | 24636.632506 |
|  | 6524.965299 | 27154.471947 |
|  | 5890.349403 | 25344.817897 |
|  | 6491.904043 | 32851.259627 |
|  | 4314.754483 | 26039.775984 |
|  | 5962.463992 | 22097.755358 |
|  | 4056.828907 | 21360.988007 |
|  | 4520.626632 | 22234.093004 |

**Raw data of soil pH**

|  | **pH** |
| --- | --- |
| CK | 5.87 |
|  | 5.88 |
|  | 5.86 |
|  | 5.60 |
|  | 5.58 |
|  | 5.60 |
|  | 5.34 |
|  | 5.35 |
|  | 5.33 |
| NPK | 5.21 |
|  | 5.19 |
|  | 5.16 |
|  | 5.06 |
|  | 5.02 |
|  | 5.02 |
|  | 4.97 |
|  | 4.93 |
|  | 4.96 |
| M30 | 5.35 |
|  | 5.35 |
|  | 5.35 |
|  | 5.54 |
|  | 5.57 |
|  | 5.52 |
|  | 5.17 |
|  | 5.15 |
|  | 5.13 |
| M50 | 5.77 |
|  | 5.75 |
|  | 5.73 |
|  | 5.73 |
|  | 5.72 |
|  | 5.73 |
|  | 5.53 |
|  | 5.39 |
|  | 5.42 |
| M70 | 6.17 |
|  | 6.16 |
|  | 6.16 |
|  | 5.80 |
|  | 5.79 |
|  | 5.74 |
|  | 5.84 |
|  | 5.85 |
|  | 5.87 |

**Table S1. Detailed information for fertilizing amount**

|  | |  | | | Early rice | | | Late rice | | |
| --- | --- | --- | --- | --- | --- | --- | --- | --- | --- | --- |
|  |  | |  | Basal fertilizer (kg/ha) | | Additional fertilizer | | Basal fertilizer (kg/ha) | Additional fertilizer | |
|  |  | |  |  |  | Tillering stage (kg/ha) | Spike stage  (kg/ha) |  | Tillering stage (kg/ha) | Spike stage  (kg/ha) |
| CK |  | | Organic fertilizer | – | | – | – | – | – | – |
|  | Mineral fertilizer | | N | – | | – | – | – | – | – |
|  |  |  | P_2_O_5_ | – | | – | – | – | – | – |
|  |  |  | K_2_O | – | | – | – | – | – | – |
| NPK |  | | Organic fertilizer | – | | – | – | – | – | – |
|  | Mineral fertilizer | | N | 75 | | 38 | 38 | 90 | 45 | 45 |
|  |  |  | P_2_O_5_ | 60 | | – | – | 60 | – | – |
|  |  |  | K_2_O | – | | 75.9 | 75.9 | – | 75.9 | 75.9 |
| M30 |  | | Organic fertilizer | 14851 | | – | – | 11790 | – | – |
|  | Mineral fertilizer | | N | 53 | | 26 | 26 | 63 | 32 | 32 |
|  |  |  | P_2_O_5_ | 42.5 | | – | – | 42.5 | – | – |
|  |  |  | K_2_O | – | | 52.7 | 52.7 | – | 52.7 | 52.7 |
| M50 |  | | Organic fertilizer | 24752 | | – | – | 19651 | – | – |
|  | Mineral fertilizer | | N | 38 | | 19 | 19 | 45 | 23 | 23 |
|  |  |  | P_2_O_5_ | 30.4 | | – | – | 30.4 | – | – |
|  |  |  | K_2_O | – | | 37.7 | 37.7 | – | 37.7 | 37.7 |
| M70 |  | | Organic fertilizer | 34653 | | – | – | 27511 | - | – |
|  | Mineral fertilizer | | N | 23 | | 11 | 11 | 27 | 14 | 14 |
|  |  |  | P_2_O_5_ | 18.2 | | – | – | 18.2 | – | – |
|  |  |  | K_2_O | – | | 22.6 | 22.6 | – | 22.6 | 22.6 |

–, No fertilizer has been added; N, nitrogen fertilizer; P_2_O_5_, phosphate fertilizer; K_2_O, potash fertilizer; CK, no fertilizer control; M30, 30% organic and 70% mineral fertilizers; M50, 50% organic and 50% mineral fertilizers; M70, 70% mineral and 30% mineral fertilizers; NPK, mineral fertilizers. The organic fertilizer applied to early rice was Chinese milk vetch, and the organic fertilizer applied to late rice was rotted pig manure.

**Table S2.** Soil chemical properties among different treatments

| **Property** | **CK** | **NPK** | **M30** | **M50** | **M70** |
| --- | --- | --- | --- | --- | --- |
| pH | 5.60 (0.23)b | 5.06 (0.10)d | 5.35 (0.17)c | 5.64 (0.15)b | 5.93 (0.18)a |
| TN (g/kg) | 1.63 (0.17)d | 2.02 (0.18)c | 2.46 (0.28)b | 2.65 (0.28)b | 3.13 (0.41)a |
| TP (g/kg) | 0.49 (0.05)e | 1.06 (0.06)d | 1.46 (0.12)c | 1.66 (0.04)b | 2.04 (0.12)a |
| TK (g/kg) | 19.2 (0.39)ab | 19.4 (0.30)a | 19.1 (0.46)ab | 19.0 (0.61)ab | 18.6 (0.58)b |
| SOC (g/kg) | 15.8 (1.44)d | 20.1 (1.99)c | 24.1 (1.98)b | 25.5 (2.07)b | 29.9 (2.66)a |
| AN (mg/kg) | 118 (15.7)d | 150 (14.9)c | 182 (32.3)b | 194 (16.3)ab | 215 (21.1)a |
| AP (mg/kg) | 4.78 (2.47)c | 48.0 (7.19)b | 88.4 (11.9)a | 92.4 (3.38)a | 100 (25.9)a |
| AK (mg/kg) | 32.9 (1.45)c | 59.3 (7.02)ab | 57.6 (1.94)b | 57.3 (3.56)b | 64.8 (4.12)a |

AN, Alkaline nitrogen; AP, available phosphorus; AK: available potassium; CK, No fertilizer control; M30, 30% organic and 70% mineral fertilizers; M50, 50% organic and 50% mineral fertilizers; M70, 70% mineral and 30% mineral fertilizers; NPK, mineral fertilizers; SOC, soil organic carbon; TK, total potassium; TN, total nitrogen; TP, total phosphorus. Different letters indicate significant differences among treatments based on a one-way analysis of variance with Tukey's HSD comparison (*P* < 0.05).

**Table S3. Pearson correlation coefficients (*r*) between soil chemical properties and abundance of *mcrA* and *pmoA* genes**

| **Property** | **Abundance** | |
| --- | --- | --- |
|  | ***mcrA*** | ***pmoA*** |
| pH | 0.598^***^ | 0.592^***^ |
| SOC | 0.676^***^ | 0.719^***^ |
| TN | 0.656^***^ | 0.724^***^ |
| TP | 0.746^***^ | 0.725^***^ |
| TK | –0.361^*^ | –0.488^***^ |
| AN | 0.558^***^ | 0.612^***^ |
| AP | 0.703^***^ | 0.605^***^ |
| AK | 0.554^**^ | 0.528^***^ |
| CH_4_ | 0.565^***^ | 0.273 |

AN, Alkaline nitrogen; AP, available phosphorus; AK, available potassium; SOC, soil organic carbon; TK, total potassium; TN, total nitrogen; TP, total phosphorus.

^*^0.01 < *P* < 0.05; ^**^0.001 < *P* < 0.01; ^***^*P* < 0.001.

**Table S4. Pearson correlation coefficients (*r*) between alpha diversity indices and soil chemical properties**

| **Pearson correlation coefficients (*n* = 45)** | | | | | | | | | |
| --- | --- | --- | --- | --- | --- | --- | --- | --- | --- |
|  | **Alpha diversity** | **pH** | **SOC** | **TN** | **TP** | **TK** | **AN** | **AP** | **AK** |
| *mcrA* | Observed OTUs | 0.231 | 0.434^**^ | 0.443^**^ | 0.471^***^ | –0.581^***^ | 0.441^**^ | 0.452^**^ | 0.257 |
|  | Shannon | 0.072 | –0.056 | −0.091 | 0.112 | –0.099 | –0.083 | 0.142 | 0.113 |
|  | Simpson | –0.036 | –0.335^*^ | –0.365^*^ | –0.171 | 0.291 | –0.384^**^ | –0.141 | –0.053 |
| *pmoA* | Observed OTUs | 0.254 | –0.292 | –0.264 | –0.247 | 0.404^**^ | –0.346^*^ | –0.245 | –0.495^***^ |
|  | Shannon | 0.713^**^ | 0.016 | 0.044 | 0.068 | 0.079 | –0.076 | –0.003 | –0.339^*^ |
|  | Simpson | 0.829^***^ | 0.380^**^ | 0.381^**^ | 0.442^**^ | –0.171 | 0.26 | 0.366^*^ | 0.071 |

AK, Available potassium; AN, alkaline nitrogen; AP, available phosphorus; SOC, soil organic carbon; TK, total potassium; TN, total nitrogen; TP, total phosphorus. ^*^0.01 < *P* < 0.05; ^**^ 0.001 < *P* < 0.01; ^***^ *P* < 0.001.

**Table S5. Analysis of similarities showing differences among community composition of methanogens and methanotrophs**

| **Treatment** | **Methanogen** | | **Methanotroph** | |
| --- | --- | --- | --- | --- |
|  | ***r*** | ***P*** | ***r*** | ***P*** |
| CK vs NPK | 0.454 | 0.001 | 0.774 | 0.001 |
| CK vs M30 | 0.663 | 0.001 | 0.347 | 0.006 |
| CK vs M50 | 0.836 | 0.001 | 0.443 | 0.001 |
| CK vs M70 | 1.000 | 0.001 | 0.984 | 0.001 |
| NPK vs M30 | 0.370 | 0.007 | 0.402 | 0.005 |
| NPK vs M50 | 0.569 | 0.001 | 0.577 | 0.001 |
| NPK vs M70 | 0.790 | 0.001 | 0.999 | 0.001 |
| M30 vs M50 | 0.271 | 0.014 | 0.017 | 0.311 |
| M30 vs M70 | 0.580 | 0.001 | 0.461 | 0.001 |
| M50 vs M70 | 0.130 | 0.053 | 0.167 | 0.046 |

CK, No fertilizer control; M30, 30% organic and 70% mineral fertilizers; M50, 50% organic and 50% mineral fertilizers; M70, 70% mineral and 30% mineral fertilizers; NPK, mineral fertilizers.


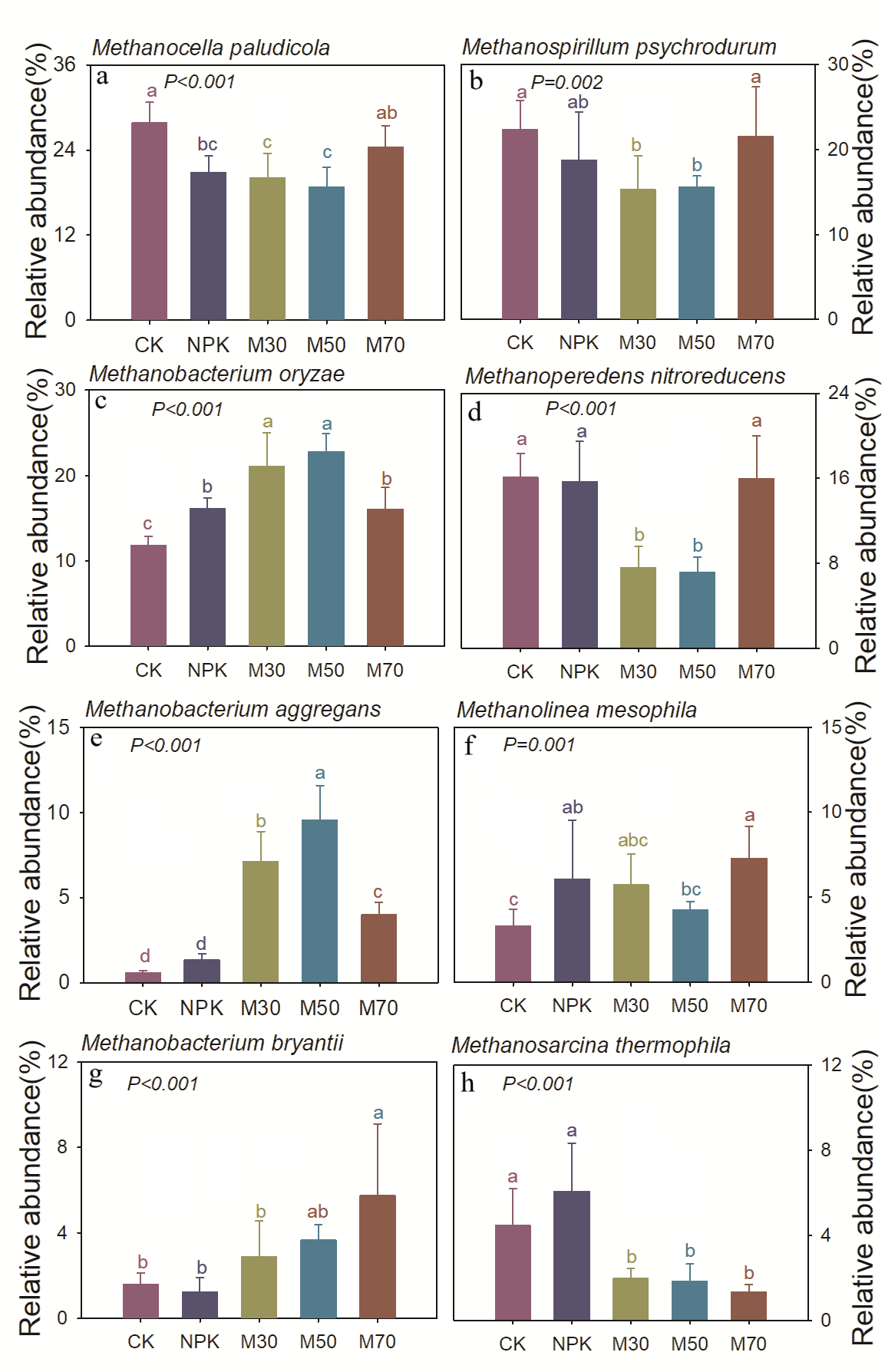


**Figure S1.** **Relative abundance of the dominant methanogenic species among treatments.** CK, No fertilizer control; M30, 30% organic and 70% mineral fertilizers; M50, 50% organic and 50% mineral fertilizers; M70, 70% mineral and 30% mineral fertilizers; NPK, mineral fertilizers.

**
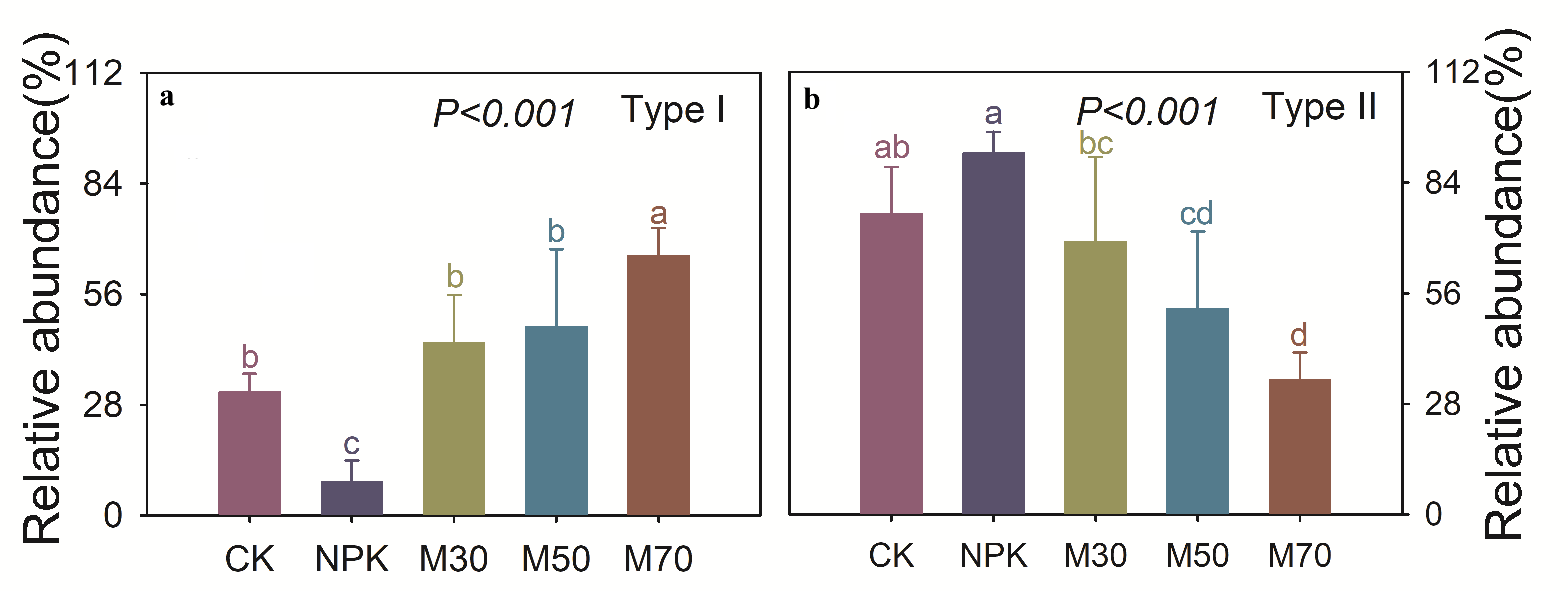
**

**Figure S2. Relative abundance of the two types of methanotrophs among treatments.** CK, No fertilizer control; M30, 30% organic and 70% mineral fertilizers; M50, 50% organic and 50% mineral fertilizers; M70, 70% mineral and 30% mineral fertilizers; NPK, mineral fertilizers.


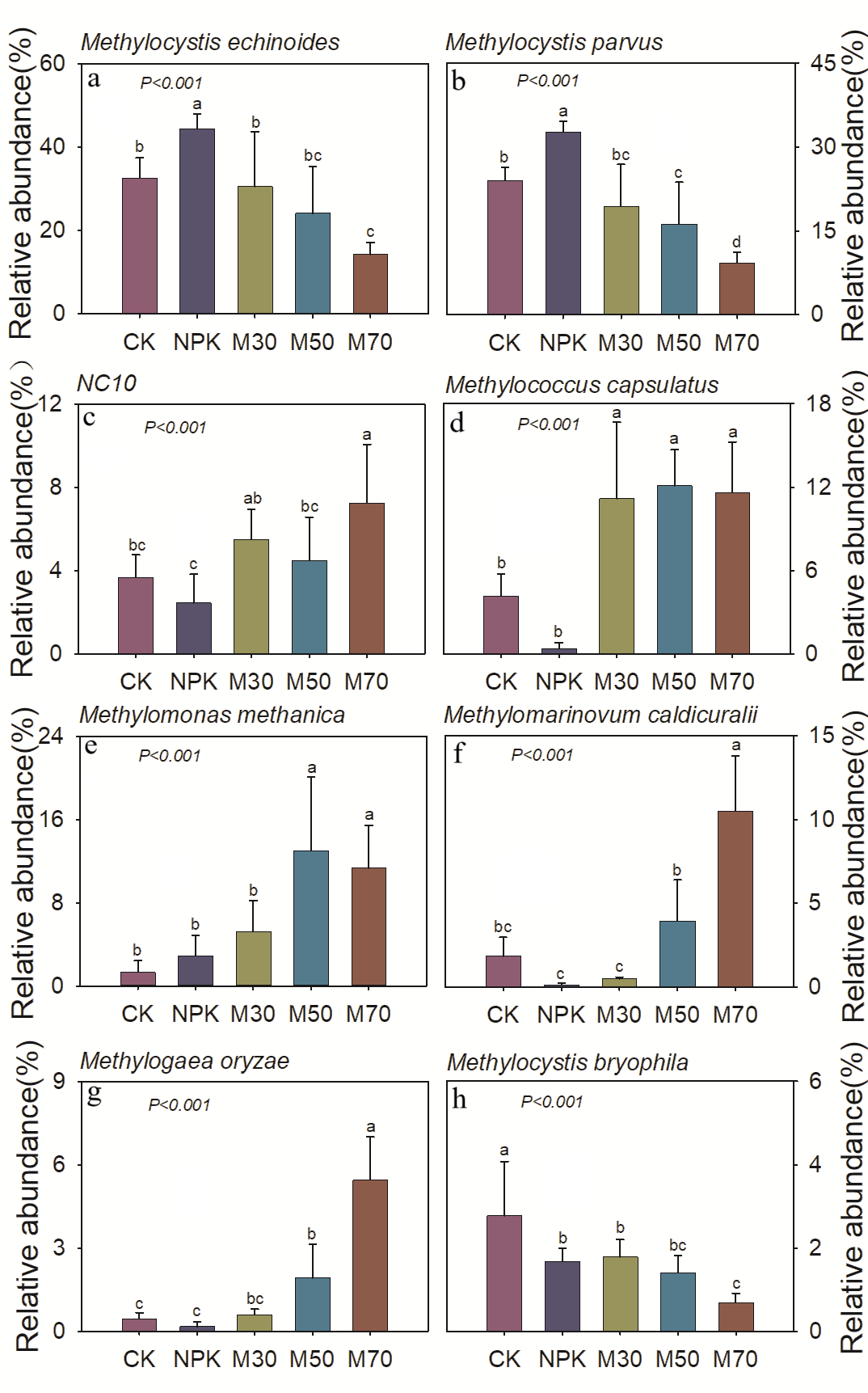


**Figure S3. Relative abundance of the dominant methanotrophic species among treatments**. CK, No fertilizer control; M30, 30% organic and 70% mineral fertilizers; M50, 50% organic and 50% mineral fertilizers; M70, 70% mineral and 30% mineral fertilizers; NPK, mineral fertilizers.
